# B cell subsets and dysfunction of regulatory B cells in IgG4-related diseases and primary Sjögren’s syndrome: the similarities and differences

**DOI:** 10.1186/ar4571

**Published:** 2014-05-29

**Authors:** Wei Lin, Lixia Jin, Hua Chen, Qingjun Wu, Yunyun Fei, Wenjie Zheng, Qian Wang, Ping Li, Yongzhe Li, Wen Zhang, Yan Zhao, Xiaofeng Zeng, Fengchun Zhang

**Affiliations:** 1 Department of Rheumatology, Peking Union Medical College Hospital, Chinese Academy of Medical Science & Peking Union Medical College, No.41 Da Mu Cang, Western District, Beijing 100032, P. R. China; 2 Tinghua University, Beijing, China

## Abstract

**Introduction:**

IgG4-related disease (IgG4-RD) is a multisystem-involved autoimmune disease. Abnormally activated and differentiated B cells may play important roles. Regulatory B cells (Breg) are newly defined B cell subgroups with immunosuppressive functions. In this study, we investigated the differences of B cell subsets, the expressions of co-stimulatory molecules on B cells, and the function of Breg cells in patients with IgG4-RD, primary Sjögren’s syndrome (pSS) as well as in healthy controls (HC).

**Methods:**

Newly diagnosed IgG4-RD patients (n = 48) were enrolled, 38 untreated pSS patients and 30 healthy volunteers were recruited as disease and healthy controls. To analyze B cell subsets and B cell activity, PBMCs were surface stained and detected by flow cytometry. The function of Breg cells was tested by coculturing isolated CD19 + CD24^hi^CD38^hi^ Breg cells with purified CD4 + CD25- T cells. Serum cytokines were measured by ELISA and cytometric bead array. Relationship between clinical data and laboratory findings were analyzed as well.

**Results:**

Compared with pSS patients and HC, IgG4-RD patients had a lower frequency of peripheral Breg cells. Interestingly, CD19 + CD24-CD38^hi^ B cell subsets were significantly higher in peripheral B cells from IgG4-RD patients than in pSS patients and HC, which correlated with serum IgG4 levels. The expression of BAFF-R and CD40 on B cells was significantly lower in IgG4-RD patients compared with those in pSS patients and HC. Unlike HC, Breg cells from pSS patients lacked suppressive functions.

**Conclusions:**

B cells in patients with IgG4-RD and pSS display a variety of abnormalities, including disturbed B cell subpopulations, abnormal expression of key signaling molecules, co-stimulatory molecules, and inflammatory cytokines. In addition, a significantly increased B cell subset, CD19 + CD24-CD38^hi^ B cells, may play an important role in the pathogenesis of IgG4-RD.

## Introduction

In recent years, a large amount of studies emphasized the status of B cells in the development of autoimmune diseases. It is well established that B cells play an inflammatory role through effective antigen presentation, production of auto-antibodies, and secretion of pro-inflammatory factors. However, B cells also produce a source of inhibitory cytokines, such as IL-10 and tumor growth factor (TGF)-β. Regulatory B cells (Breg), a group of new B cell members with the ability to inhibit the immune response, play an important role in maintaining the balance and tolerance in immune function [1-4].

IgG4-related disease (IgG4-RD) is a newly recognized systemic inflammatory condition characterized by tumefactive lesions, elevated serum IgG4 levels (>135 mg/dl), and IgG4+ plasma cell infiltration (IgG4+ cells in tissue account for more than 40% of the total number of plasma cells) [5]. The disease can affect multiple organs or tissues, such as the lacrimal gland, submandibular gland, pancreas, retroperitoneal tissue, and the bile duct, resulting in swelling and sclerosis of the involved organs. The complications of IgG4-RD include Mikulicz’s disease (MD), autoimmune pancreatitis, retroperitoneal fibrosis, tubulointerstitial nephritis, and Riedel’s thyroiditis, *et al*. Previous studies have found that Th2 cells are predominantly activated at affected sites. Collected peripheral blood mononuclear cells (PBMCs) from IgG4-RD patients principally produce Th2-type cytokines, which indicates that a Th1-Th2 imbalance may play a crucial role in IgG4-RD [6,7]. However, the role of B cells in the pathogenesis of IgG4-RD remains unclear.

Primary Sjögren’s syndrome (pSS) is a systemic autoimmune disease affecting the exocrine glands, primarily the lacrimal and salivary glands, resulting in dryness of the eyes and mouth [8]. There are several similarities between IgG4-RD and pSS. First, both diseases present with hypergammaglobulinemia. Next, in both diseases, the characteristic features of involved tissues are massive lymphocyte infiltration or even generation of germinal centers. Furthermore, both diseases are systemic with multiple organ involvement, especially lacrimal and salivary glands. MD, a singular IgG4-RD, was considered part of pSS for a long time [9]. Finally, both diseases respond well to corticosteroid therapy.

To date, the role of Breg cells has been identified in the regulation of several immune-mediated processes of different disease types, such as autoimmune diseases [10-12], infectious disease [13], and cancer [14]. It has been demonstrated that human peripheral CD19 + CD24^hi^CD38^hi^ Breg cells possess regulatory function in immune responses. Given abnormal B cells are generally considered as one of key mechanisms of pSS, we hypothesize that B cell abnormality participates in the pathogenesis of IgG4-RD. In this study, we evaluated the peripheral B cell subsets, especially Breg cells in IgG4-RD patients, pSS patients, and HC, as well as B cell co-stimulatory and activation markers of CD80, CD86, CD40, and B cell activating factor receptor expression. The correlations of B cell subsets with clinical features were analyzed as well. The immune regulatory function of Breg cells was explored in order to explain the role(s) of Breg in the pathogenesis of IgG4-RD.

## Materials and methods

### Patients and controls

Untreated patients with IgG4-RD (n = 48), untreated patients with pSS (n = 38), and healthy controls (HC) (n = 30) were enrolled in this study. Patients with IgG4-RD and pSS fulfilled the 2011 comprehensive IgG4-RD diagnostic criteria [15], and the 2002 European/American consensus group diagnostic criteria [16], respectively. The diagnosis of IgG4-RD was based on the following three items: 1) clinical examination showing characteristic diffuse/localized swelling or masses in single or multiple organs; 2) hematological examination showing elevated serum IgG4 concentration (>135 mg/dl); 3) histopathologic examination showing: (A) marked lymphocytes and plasma cells infiltration and fibrosis, or (B) infiltration of IgG4+ plasma cells (ratio of IgG4+/IgG + cells >40% and >10 IgG4+ plasma cells/high power field). Patients with cancer or lymphoma and other autoimmune diseases were excluded. In this study, only those untreated pSS patients with hypergammaglobulinemia were included. Age-matched healthy individuals were studied in parallel as controls. The study was approved by the Medical Ethics Committee of Peking Union Medical College Hospital (Peking, China). Written informed consent was obtained from all patients and HC.

### Phenotypic analysis of peripheral B cells by flow cytometry

Peripheral blood mononuclear cells (PBMCs) from IgG4-RD, pSS, and HC were prepared according to standard Ficoll-Hypaque procedures (Pharmacia Biotech, Uppsala, Sweden), and were stained with PE-CY7-anti-CD19, FITC-anti-CD24, APC-anti-CD38, PE-anti-CD40, PE-anti-CD80, PE-anti-CD86, PE-anti-BAFF-R, PE-anti-TACI mAb (BD Biosciences, San Jose, CA, USA), and PE-anti-BCMA mAb (Biolegend, San Diego, CA, USA) or isotype-matched controls. After incubation for 30 minutes at 4°C, the cells were washed and resuspended in fluorescence-activated cell sorter (FACS) staining buffer (BD Biosciences). All experiments were analyzed by gating on lymphocytes according to forward side scatter/side light scatter; dead or dying cells or granulocytes were excluded. B-cell subsets were gated on CD19, and then gated on CD24 and CD38 [17]. Flow cytometry analysis was performed immediately after sample preparation. All samples were analyzed using a FACS AriaII (BD Biosciences)and data were analyzed by FlowJo v.7.6.4 Software (Tree Star, Stanford University, CA, USA).

### Cell isolation and culture

#### Cell isolation

PBMCs were stained with CD19, CD24, and CD38 antibodies, and then CD19 + CD24 + CD38-memory B cells and CD19 + CD24^hi^CD38^hi^ Breg cells were sorted with a MoFlo high-performance cell sorter (Cytomation, Fort Collins, CO, USA). CD4 + CD25- effector T cells were purified by magnetic-bead separation with MACS kits (Miltenyi, Gladbach, Germany) according to the manufacturer’s instructions, achieving >95% purity.

#### Cell culture

To test the function of Breg cells, co-cultured cells (Breg cells and CD4 + CD25- T cells from patients or healthy donors at a ratio of 1:1, 5*10^4^ cells/well) were resuspended in Roswell Park Memorial Institute (RPMI) 1640 medium supplemented with 10% FCS and antibiotics (penicillin 100 IU/mL, streptomycin 100 μg/mL; Invitrogen, Camarillo, CA, USA) in 96-well U-bottom plates (Nunc, Langenselbold, Germany) in a humidified atmosphere of 5% CO_2_ at 37°C, in the presence of 0.5 μg/mL purified plate-bound CD3 monoclonal antibody, 100 ng/mL recombinant human CD40L (Abcam, Cambridge, MA, USA), 0.1 μg/mL cytosine-phosphate-guanosine oligo-deoxynucleotide 2006 (CpG ODN 2006, Invivogen, San Diego, CA, USA). From the same healthy donor, 100,000 PBMCs depleted of CD4+ T cells by magnetic-bead purification, were incubated with 25 μg/mL mitomycin C at 37°C for 30 minutes, and were added to co-cultures as feeder cells.

For the experiment with memory B cells, purified CD19 + CD24 + CD38- memory B cells from IgG4-RD, pSS patients and HC were resuspended in RPMI1640 medium supplemented with 10% FCS and antibiotics in 96-well U-bottom plates in a humidified atmosphere of 5% CO2 at 37°C with 1 × 10^5^ in each well. For each group, 100 ng/mL recombinant human CD40L (Abcam) and 0.1 μg/mL CpG ODN 2006 (Invivogen) was added at the beginning.

To test the effect of BAFF-induced BAFF-R expression, PBMCs were cultured with or without rhBAFF (R&D Systems, Minneapolis, MN, USA), at the concentration similar to the range observed in the serum of patients with IgG4-RD. Cells were cultured for 48 h, then BAFF-R expression on B cells was analyzed by FACS gating on live lymphocytes.

### Intracellular cytokine analysis

For intracellular cytokine measurement, cells were incubated with PMA (20 ng/mL, Sigma-Aldrich, St Louis, MO, USA), ionomycin (1,000 ng/mL, Sigma-Aldrich) and GolgiPlug (1ul/mL, BD Biosciences) for the last 5 h. After surface staining with CD4-PerCP-CY5.5, cells were washed, fixed, permeabilized, and stained with interferon (IFN)-γ-APC and TNF-α-APC mAbs (BD Biosciences). Appropriate APC-conjugated isotype controls were used for gate setting of cytokine expression.

### Enzyme-linked immunosorbent assay (ELISA) and cytometric bead array (CBA) analysis

Serum samples from enrolled patients and controls were collected and were stored at -80°C until used. Serum cytokine levels of IgG4-RD, pSS, and HC were measured by human ELISA kits (R&D Systems) according to the manufacturer’s instructions. CBA analysis for IgG4 in supernatants was performed according to the manufacturer’s instructions (BD Biosciences). Data were analyzed using CBA analysis software from BD Biosciences. The concentration of IgG4 in supernatants was determined by reference to a standard curve.

### Clinical data and inflammatory parameters

Clinical data including age, gender, disease duration, and manifestations were reported in all patients. Laboratory findings were recorded, including erythrocyte sedimentation rate (ESR), C-reactive protein (CRP), serum immunoglobulin (Ig)G, IgA, IgM, and IgG subsets.

### Statistical analysis

Results were summarized as mean ± SD and standard error of the mean (SEM). Quantitative comparisons involved the Mann-Whitney U test for unpaired data and the Wilcoxon test for paired data. The relationship between frequency levels of B-cell subsets and clinical features in IgG4-RD and pSS patients were analyzed by Pearson’s rank test. All statistical analyses were performed using SPSS v.17.0 statistics package software (IBM, USA) and GraphPad Prism software version 5.0 (Graph-Pad, San Diego, CA, USA). A *P*-value <0.05 was considered significantly different.

## Results

### Patient characteristics

Demographic, clinical, and laboratory characteristics of enrolled IgG4-RD patients, pSS patients, and HC are summarized in Table 1. There was no statistical difference in total serum IgG levels between IgG4-RD (24.58 ± 10.74 g/L) and pSS patients (22.99 ± 11.48 g/L; *P* >0.05); however, the serum IgA and IgM levels in IgG4-RD patients (1.85 ± 0.76 g/L, 0.82 ± 0.38 g/L, respectively) were significantly lower compared with those in pSS patients (4.17 ± 2.23 g/L; *P* <0.001 and 1.24 ± 0.64 g/L; *P* = 0.001). Serum IgG4 levels were remarkably elevated in IgG4-RD patients (1929.10 ± 1923.63 mg/dL), significantly higher than in pSS patients (36.45 ± 37.74 mg/dL; *P* <0.001). Furthermore, the ratio of IgG4/ IgG was significantly increased in IgG4-RD patients.

**Table 1:** Clinical and laboratory findings in IgG4-related disease, primary Sjögren’s syndrome and healthy controls

	**Healthy controls**	**IgG4-related disease**	**Primary Sjögren’s syndrome**
**Number**	30	48	38
**Gender, male/female, n**	7/23	29/19	3/35
**Age, years**	46.4 ± 10.0	53.17 ± 13.66***	42.2 ± 9.6
**Disease duration, months**	NA	29.45 ± 41.35*	51.32 ± 47.6
**ESR, mm/h**	NA	49.35 ± 33.80**	30.68 ± 28.35
**C-reactive protein, mg/L**	NA	9.17 ± 3.67**	2.65 ± 1.64
**Immunoglobulin (Ig)G, g/L**	10.11 ± 1.47	24.58 ± 10.74	22.99 ± 11.48
**IgA, g/L**	2.28 ± 0.74	1.85 ± 0.76***	4.17 ± 2.23
**IgM, g/L**	1.02 ± 0.50	0.82 ± 0.38**	1.24 ± 0.64
**IgG1, mg/dL**	797.83 ± 106.54	1036.55 ± 670.09**	1485.74 ± 486.36
**IgG2, mg/dL**	554.6 ± 228.80	592.76 ± 396.55**	372.95 ± 126.70
**IgG3, mg/dL**	41.68 ± 22.10	78.61 ± 64.05	60.03 ± 41.41
**IgG4, mg/dL**	57.06 ± 46.06	1929.10 ± 1923.63***	36.45 ± 37.74
**IgG4/total IgG%**^**1**^	3.90 ± 3.10	39.03 ± 25.84***	1.86 ± 1.85

Values are shown as mean ± SD unless stated otherwise. ^1^IgG level is the sum of IgG1, IgG2, IgG3 and IgG4. ***P <0.001; **P <0.01; *P <0.05 (compared with Primary Sjögren’s syndrome). ESR, erythrocyte sedimentation rate; NA, not applicable.

### Decreased regulatory and mature but increased memory B cells in IgG4-RD patients

**Figure 1:**
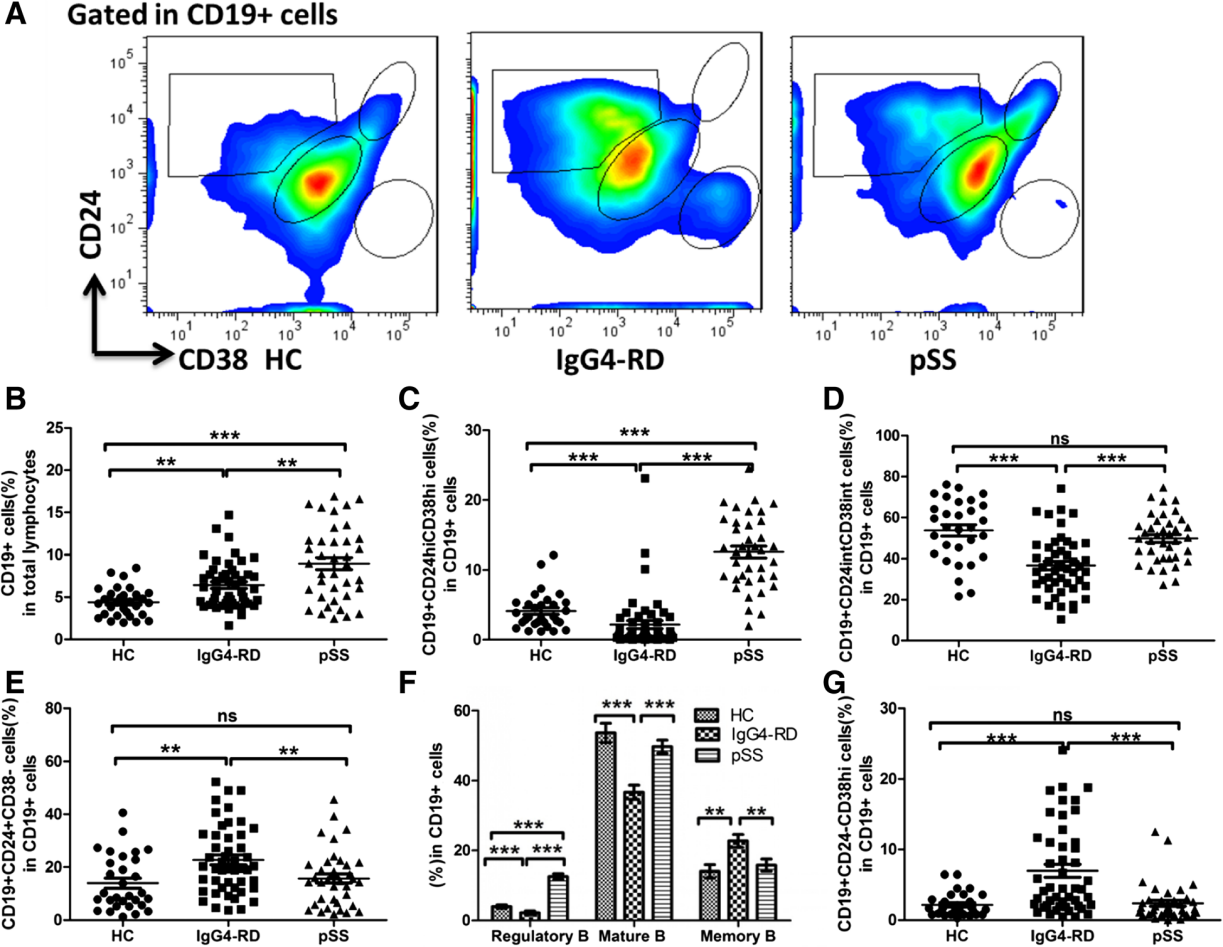
**Expression of B-cell subsets in IgG4-related disease (RD), primary Sjögren’s syndrome (pSS), and healthy controls (HC).** Representative flow cytometry pictures of different B-cell subsets from HC, IgG4-RD, and pSS patients **(A)**. The percentages of CD19+ B cells out of total lymphocytes in each group **(B)**. Percentages of Breg cells, mature B cells, and memory B cells out of total B cells in each group **(C, D, E)**. Summary of different B-cell subsets in different populations **(F)**. Percentages of CD19 + CD24-CD38^hi^ B cells out of total B cells in each group **(G)**. Values are shown as mean ± standard error of the mean, *P <0.05, **P <0.01, ***P <0.001.

In order to evaluate possible changes in B-cell populations in IgG4-RD and pSS patients, we compared the percentages of total, regulatory, mature, and memory B cells in peripheral blood. According to previous reports [11,17-19], B cell subsets were briefly defined as mature (CD19 + CD24^int^CD38^int^), memory (CD19 + CD24 + CD38-) and regulatory (CD19 + CD24^hi^CD38^hi^) B cells (Figure 1A).

The percentages of CD19+ B cells were significantly increased in IgG4-RD patients (6.43 ± 2.73%) compared to HC (4.41 ± 1.75%; *P* = 0.001), but were lower than in pSS patients (8.97 ± 4.40%; *P* <0.001; Figure 1B). The median fluorescence intensity (MFI) of CD19+ B cells was significantly different among three groups (HC: 145.50 ± 27.62; IgG4-RD: 207.9 ± 65.50; pSS: 259.80 ± 90.79; *P* <0.001).

The frequency of regulatory B cells in IgG4-RD patients was lower compared with pSS patients and HC (2.17 ± 3.96%, 12.55 ± 5.15%, and 3.98 ± 2.70%, respectively; *P* <0.001; Figure 1C). Moreover, there were decreased percentages of mature B cells in IgG4-RD patients compared with pSS patients and HC (36.68 ± 14.27%, 49.75 ± 11.59%, and 53.70 ± 15.12%, respectively; *P* <0.001; Figure 1D). In contrast, IgG4-RD patients had increased percentages of memory B cells compared with HC and pSS patients (22.71 ± 12.81%, 14.01 ± 10.39%, and 15.79 ± 10.58%, respectively; *P* <0.01; Figure 1E). Figure 1F summarizes the percentages of B-cell subsets in the IgG4-RD, pSS, and HC.

Interestingly, an undefined CD19 + CD24-CD38^hi^ B-cell population was significantly increased in IgG4-RD patients (6.99 ± 6.24%) compared with those from pSS (2.39 ± 2.64%; *P* <0.001) and HC (2.16 ± 1.65%; *P* <0.001; Figure 1G). Table 2 summarizes the proportions of B-cell subsets in IgG4-RD, pSS and HC.

**Table 2:** B cell phenotypes in IgG4-RD, pSS and healthy controls

	**Healthy controls**	**IgG4-RD**	**pSS**	**IgG4-related disease versus HC**	**pSS versus HC**	**IgG4-RD versus pSS**
				***P*-value**	***P*-value**	***P*-value**
CD19+ (%)^1^	4.41 ± 1.75	6.43 ± 2.73	8.97 ± 4.40	0.001	<0.001	0.009
CD19 + CD24 + CD38- (%)^2^	14.01 ± 10.39	22.71 ± 12.81	15.79 ± 10.58	0.002	0.541	0.009
CD19 + CD24intCD38int (%)^2^	53.70 ± 15.12	36.68 ± 14.27	49.75 ± 11.59	<0.001	0.150	<0.001
CD19 + CD24hiCD38hi (%)^2^	3.98 ± 2.70	2.17 ± 3.96	12.55 ± 5.15	<0.001	<0.001	<0.001
CD19 + CD24-CD38hi (%)^2^	2.16 ± 1.65	6.99 ± 6.24	2.39 ± 2.64	<0.001	0.868	<0.001
CD19 + CD40+ (%)^2^	87.08 ± 11.27	44.35 ± 21.79	69.48 ± 20.33	<0.001	<0.001	<0.001
CD19 + BAFF-R + (%)^2^	94.13 ± 3.50	76.63 ± 20.85	88.96 ± 8.90	0.001	0.005	0.066
CD19 + CD80+ (%)^2^	17.54 ± 7.41	43.27 ± 8.64	23.69 ± 14.24	<0.001	0.327	0.037
CD19 + CD86+ (%)^2^	19.56 ± 7.70	47.18 ± 14.23	27.45 ± 12.17	0.002	0.325	0.041

Values are shown as mean ± SD. ^1^Percentages of CD19+ cells in total lymphocytes. ^2^Percentages of B cell subsets in CD19+ cells. IgG4-RD, IgG4-related disease; pSS, primary Sjögren’s syndrome.

In addition, after one to three months of glucocorticoids treatment, the ratio of CD19 + CD24-CD38^hi^ B cells decreased to 2.54 ± 2.28% in IgG4-RD patients (*P* = 0.01), whereas there were no statistically significant changes in other B-cell subgroups.

### Decreased expression of BAFF-R on peripheral B-cell subsets in IgG4-RD patients

**Figure 2:**
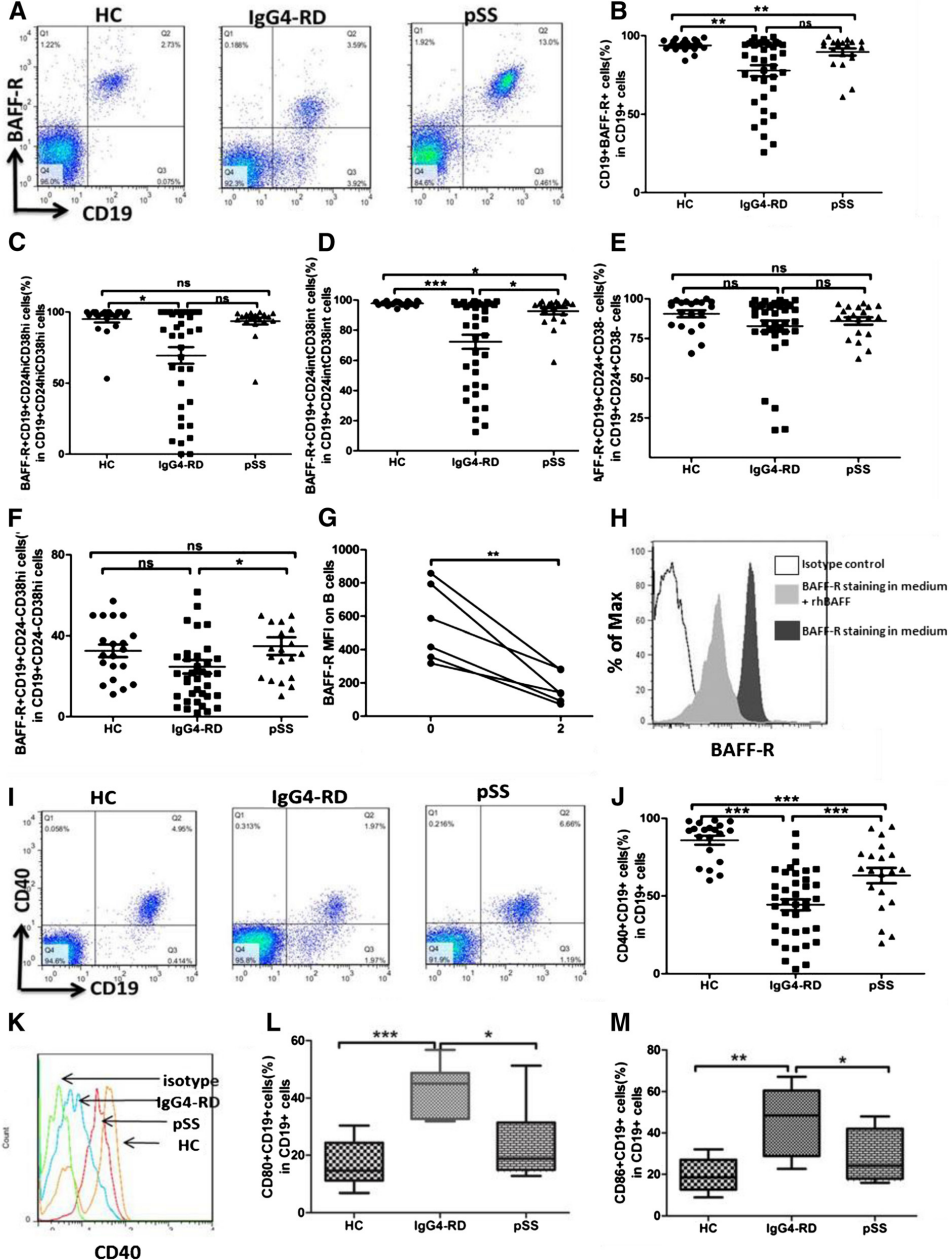
**Expression of BAFF-R, CD40, CD80, and CD86 on peripheral B cells in IgG4-related disease (RD), primary Sjögren’s syndrome (pSS), and healthy controls (HC).** Representative flow cytometry pictures of BAFF-R expression on CD19+ B cells of HC, IgG4-RD, and pSS patients **(A)**. Scatter plots showing the percentages of CD19 + BAFF-R + cells in CD19+ cells **(B)**. Scatter plots of the percentages of BAFF-R + expression on different B cell subsets **(C-F)**. The changes in mean fluorescence intensity (MFI) of BAFF-R from healthy subjects after stimulation with recombinant human BAFF (rhBAFF) for 48 h **(G)**. One representative example of decreased BAFF-R MFI on B cells by flow cytometry after 48 h culture in the presence of rhBAFF (2 ng/mL) **(H)**. Representative flow cytometry of CD40 expression on CD19+ B cells of HC, IgG4-RD, and pSS patients **(I)**. Scatter plots showing the percentages of CD19 + CD40+ cells in CD19+ cells **(J-K)**. Percentages of CD80 and CD86 expression on CD19+ B cells of HC, IgG4-RD, and pSS patients **(L,M)**. Values are shown as mean ± standard error of the mean, *P <0.05; **P <0.01; ***P <0.001.

BAFF-R expression was tested in 38 IgG4-RD patients, 20 pSS patients and 20 HC. The frequency of BAFF-R expression on B cells was significantly lower in IgG4-RD patients compared with HC (76.63 ± 20.85% and 94.13 ± 3.50%; *P* <0.01), but was comparable to pSS patients (88.96 ± 8.90%; *P* = 0.066; Figure 2A, B). Moreover, MFI of BAFF-R in CD19+ B cells from IgG4-RD patients was lower than in HC (243.9 ± 149.35 and 430.56 ± 109.28, respectively; *P* <0.001), but was similar to that in pSS patients (323.5 ± 96.46; *P* >0.05).

As B-cell subpopulation homeostasis was disturbed in IgG4-RD and pSS, we analyzed BAFF-R expression in different B-cell subsets. The percentages of BAFF-R + Breg cells were significantly lower in IgG4-RD patients compared with HC (69.47 ± 35.31%, 94.66 ± 11.23%, respectively; *P* = 0.01), while there was no significant difference compared with pSS patients (93.58 ± 10.69%; *P* = 0.15; Figure 2C). The MFI of BAFF-R in Breg cells of IgG4-RD patients was lower than that of HC (208.5 ± 218.4, 344.1 ± 158.4, respectively; *P* <0.05) and pSS patients (340.4 ± 114.5; *P* <0.05). The proportions of BAFF-R + mature B cells were significantly decreased in IgG4-RD patients compared with pSS patients and HC (72.27 ± 28.91%, 92.59 ± 92.7%, and 97.78 ± 1.51%, respectively; *P* <0.05; Figure 2D). Meanwhile, the MFI of BAFF-R on mature B cells of IgG4-RD patients (193.1 ± 180.9) was lower than that of pSS patients (374.0 ± 136.5; *P* = 0.02) and HC (465.5 ± 109.2; *P* <0.001), whereas there was no significant difference between pSS patients and HC. Regarding the percentages of BAFF-R expression on memory B cells, there was no difference among IgG4-RD, pSS, and HC (IgG4-RD: 82.78 ± 21.63%; pSS: 86.06 ± 10.35%; HC: 90.21 ± 10.08%; *P* = 0.30; Figure 2E). Similarly, the MFI of BAFF-R on memory B cells was not statistically different in the three groups (IgG4-RD: 280.1 ± 124.2; pSS: 286.8 ± 101.0; HC: 364.2 ± 140.7; *P* = 0.10).

The percentages of BAFF-R + CD19 + CD24-CD38^hi^ B cells were significantly decreased in IgG4-RD compared with pSS patients (23.29 ± 20.83%, 34.91 ± 19.65%, respectively; *P* = 0.045), and were comparable to HC (32.31 ± 14.26%; *P* = 0.097; Figure 2F). No significant differences in the MFI of BAFF-R on CD19 + CD24-CD38^hi^ B cells were found among three groups (IgG4-RD: 11.37 ± 13.62; pSS: 13.39 ± 9.79; HC: 11.43 ± 5.32).

In the meantime, the expression of other two kinds of BAFF receptors, B-cell maturation antigen (BCMA) and transmembrane activator and calcium modulator (TACI) were tested, which showed no significant difference among IgG4-RD, pSS and HC. The frequency of BCMA expression on B cells was comparable in IgG4-RD patients and pSS patients and HC (5.19 ± 1.48%, 5.80 ± 3.29%, and 5.42 ± 1.78%, *P* = 0.97; respectively). Similarly, the frequency of TACI on B cells was not statistically different among the three groups (IgG4-RD: 5.56 ± 1.63%; pSS: 5.33 ± 1.59%; HC: 4.69 ± 0.51%; *P* = 0.82).

To determine the response of BAFF-R to BAFF stimulation, PBMC from healthy subjects were incubated with or without rhBAFF at a concentration similar to the range observed in the serum of patients with IgG4-RD for 48 h. The MFI of BAFF-R decreased with stimulation of rhBAFF (from 554.80 ± 230.2 to 166.8 ± 92.66; *P* <0.01; Figure 2G-H), indicating that expression of BAFF-R might be inhibited by chronic BAFF overproduction in IgG4-RD patients.

### Decreased expression of CD40 but increased expression of CD80 and CD86 on peripheral B cell subsets in IgG4-RD patients

CD40, CD80 and CD86 expressions were tested in 38 IgG4-RD patients, 20 pSS patients and 20 HC. Both the percentage and the MFI of CD40 on CD19+ B lymphocytes were lower in IgG4-RD patients (44.35 ± 21.79%, 12.32 ± 6.28, respectively) compared with those in pSS patients (69.48 ± 20.33%; *P* <0.001 and 21.47 ± 8.23; *P* <0.001; respectively) and in HC (87.08 ± 11.27%; *P* < 0.001 and 27.32 ± 6.13; *P* <0.001; respectively; Figure 2I-J). Analysis of MFI revealed that CD40 expression on both regulatory and mature B cells was lower in IgG4-RD patients (14.28 ± 8.91, 12.70 ± 7.14, respectively) compared with those in pSS patients (24.13 ± 6.06; *P* <0.001 and 26.09 ± 6.90; *P* <0.001; respectively) and in HC (20.75 ± 5.72; *P* <0.05 and 23.93 ± 5.86; *P* <0.001; respectively). The MFI of CD40 on memory B cells was significantly lower in IgG4-RD than in pSS patients (16.48 ± 6.68, 25.55 ± 13.63, respectively; *P* <0.05).

Next we evaluated the expression of co-stimulatory molecules of B cells, CD80 and CD86. The results revealed that CD19+ B lymphocytes in IgG4-RD patients expressed significantly higher CD80 and CD86 (43.27 ± 8.64%, 47.18 ± 14.23%, respectively) than in pSS patients (23.69 ± 14.24%; *P* = 0.037 and 27.45 ± 12.17%; *P* = 0.041; respectively) and HC (17.54 ± 7.41%; *P* <0.001 and 19.56 ± 7.70%; *P* = 0.002, respectively; Figure 2K-M).

### Breg cells are deficient in inhibition of proinflammatory cytokine production by T cells in pSS patients

**Figure 3:**
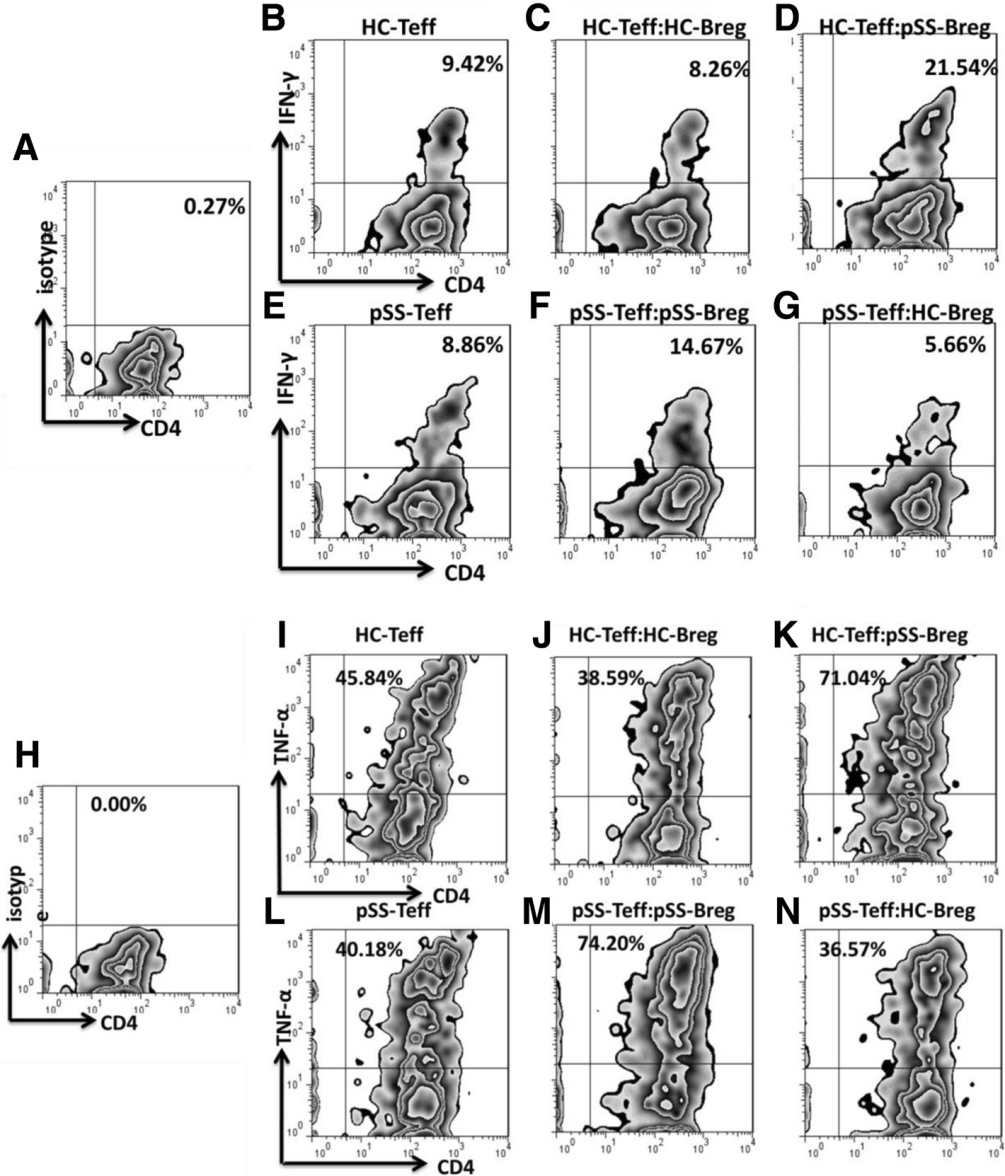
**Inhibiting function of Breg cells in primary Sjögren’s syndrome (pSS) patients.** Magnetic-bead purified CD4 + CD25- effector T cells were cultured alone or 1:1 with flow-cytometry sorted CD19 + CD24^hi^CD38^hi^ cells, stimulated for 72 h with 0.5 μg/mL plate-bound CD3 monocolonal antibodies (mAb), 100 ng/mL CD40L and 0.1 μg/mL CpG ODN 2006. GolgiPlug was added during the final 5 h along with PMA + Iono. Cells were surface-stained for the expression of CD4 and intracellular-stained IFN-γ and TNF-α. Expression of IFN-γ and TNF-α were assessed by flow cytometry. Isotype-matched mAbs were set to be negative controls **(A, H)**. Flow cytometry plots of IFN-γ and TNF-α expression by effector T cells (Teff) alone **(B, E, I, L)** or in the presences of CD19 + CD24^hi^CD38^hi^ B cells are shown **(C, F, J, M)**. Heterologous Teff/Breg cells from HC and Breg/Teff cells from pSS patients were cross-cultured at 1:1 in the same condition. The results are shown in **D**, **G**, **K**, **N**. Data are representative of six independent experiments (six different donors in each group).

To assess the function of regulatory B cells in pSS patients, magnetic-bead-purified CD4 + CD25-effector T cells (Teff) were cultured alone or 1:1 with flow-cytometry sorted CD19 + CD24^hi^CD38^hi^ cells, stimulated for 72 h with 0.5 μg/mL plate-bound CD3 mAb, 100 ng/mL CD40L and 0.1 μg/mL CpG ODN 2006. GolgiPlug was added during the final 5 h along with PMA (20 ng/mL) and ionomycin (1000 ng/mL). Cells were surface-stained for the expression of CD4 and intracellular stained for IFN-γ and TNF-α. The expression of IFN-γ and TNF-α was evaluated by flow cytometry. Isotype-matched mAbs were set as negative controls (Figure 3A, H). The results revealed that, in HC, co-culture of CD4 + CD25- T cells with CD19 + CD24^hi^CD38^hi^ B cells suppressed the frequencies of CD4 + IFN-γ + (Figure 3C) and CD4 + TNF-α + (Figure 3J) cells compared with effector T cells alone (Figure 3B, I). In contrast, compared with effector T cells alone (Figure 3E, L), CD19 + CD24^hi^CD38^hi^ Breg cells from pSS patients did not show effects of suppressing proinflammatory cytokines by CD4 + CD25- effector T cells (Figure 3F, M).Next, we evaluated whether Teff cells from pSS patients were resistant to the suppressive effect of Breg cells or whether there was malfunction of Breg cells. Heterologous Teff/Breg cells from HC and Breg/Teff cells from pSS patients were cross-cultured at 1:1 in the same condition. The results demonstrated that IFN-γ and TNF-α expressed by pSS Teff cells could be suppressed by healthy Breg cells, indicating that the suppressive function of Breg cells from pSS patients was deficient (Figure 3D, G, K, N).

The immunoregulatory function of Breg cells from IgG4-RD was not tested because of low frequency. In addition, isolated B cells from IgG4-RD patients were stimulated with CD40L and CpG ODN 2006 for 3, 6, and 9 days, but no induced increase of Breg cells (data not shown).

### CD19 + CD24 + CD38- memory B cells from IgG4-RD patients secreted more IgG4

**Figure 4:**
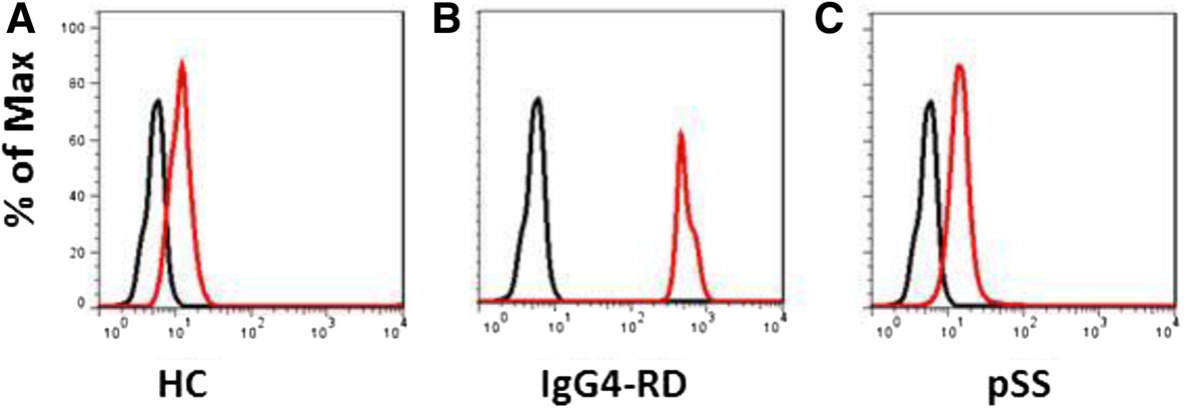
**IgG4 secreting by CD19 + CD24 + CD38- memory B cells from IgG4-related disease patients.** Isolated CD19 + CD24 + CD38- memory B cells from IgG4-RD, primary Sjögren’s syndrome (pSS) patients and healthy controls (HC) were cultured *in vitro*, stimulated with CD40L and CpG ODN 2006. At day 7, the supernatants were collected and IgG4 level was evaluated by CBA. The results are shown in **A**-**C**.

CD19 + CD24 + CD38- memory B cells from IgG4-RD, pSS patients and HC were isolated and cultured *in vitro*, with CD40L and CpG ODN 2006. At day 7, the culture supernatants were collected and IgG4 level was evaluated by CBA. The results showed that memory B cells from IgG4-RD patients secreted more IgG4 (IgG4-RD: 161.00 ± 55.91 ng/mL; HC: 6.19 ± 2.40 ng/mL; pSS: 5.86 ± 3.15 ng/mL, respectively; Figure 4).

### Serum cytokine levels and BAFF levels in IgG4-RD, pSS, and HC

**Figure 5:**
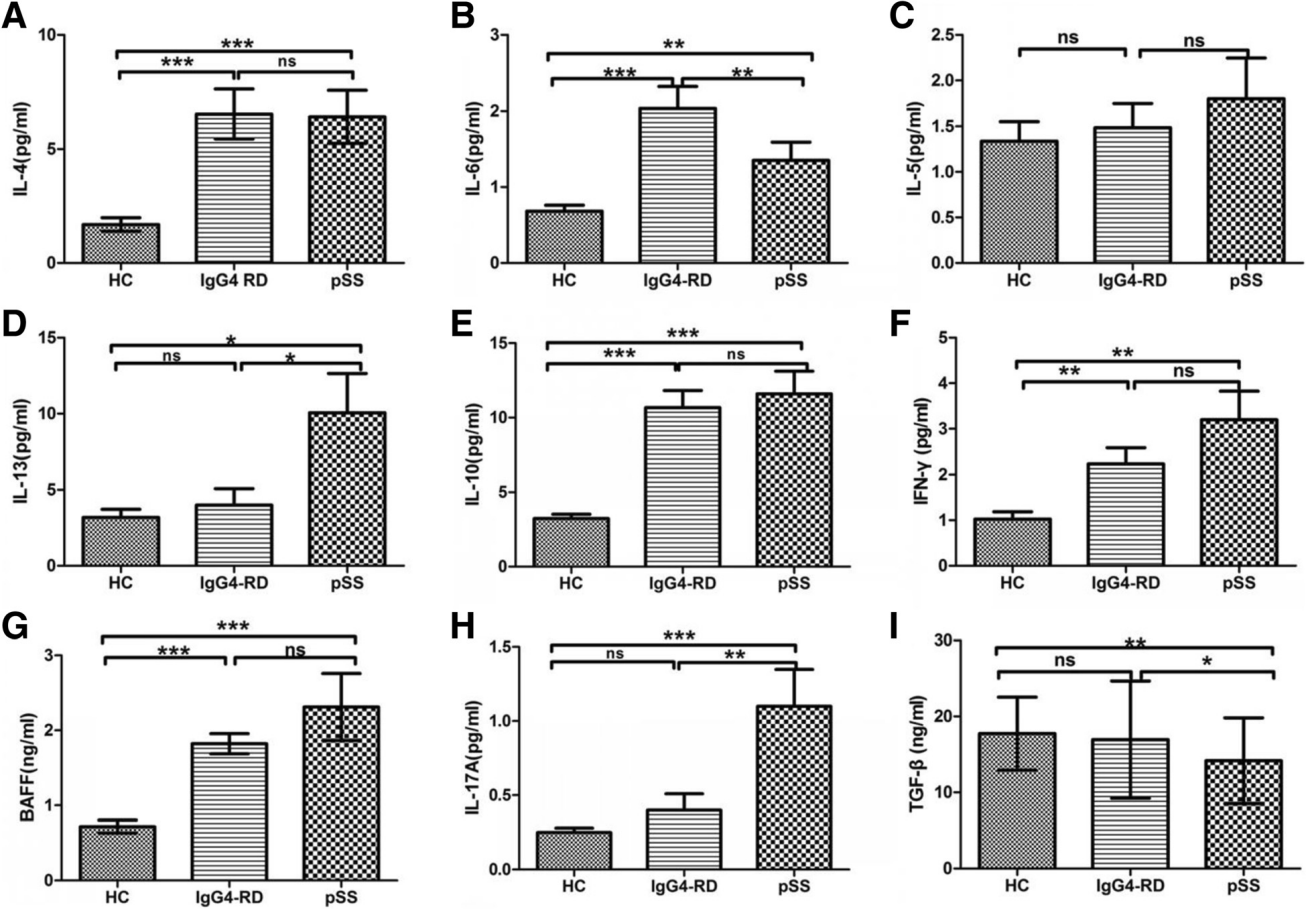
**Serum levels of cytokines and BAFF in IgG4-related disease (RD), primary Sjögren’s syndrome (pSS), and healthy controls (HC).** Serum cytokines of IL-4 **(A)**, IL-6 **(B)**, IL-5 **(C)**, IL-13 **(D)**, IL-10 **(E)**, IFN-γ **(F)**, IL-17A **(H)**, TGF-β **(I)** and BAFF **(G)** levels from serum samples of IgG4-RD (n = 28), pSS patients (n = 28), and HC (n = 24) were tested by ELISA. Values are shown as mean ± standard error of the mean, *P <0.05; **P <0.01; ***P <0.001.

IgG4-RD patients (n = 28), pSS patients (n = 28), and HC (n = 24) were tested for serum cytokine profile. Serum levels of IL-4 in patients with IgG4-RD (6.54 ± 5.67 pg/mL) were significantly higher than those in HC (1.69 ± 1.46 pg/mL; *P* <0.001), and there were no significant differences between IgG4-RD and pSS patients (6.52 ± 6.14 pg/mL) (Figure 5A), Serum levels of IL-6 in patients with IgG4-RD (2.14 ± 1.66 pg/mL) were significantly higher than those in HC and pSS patients (0.68 ± 0.39 pg/mL, *P* <0.001; 1.35 ± 1.40 pg/mL; *P* = 0.002; Figure 5B). Serum levels of IL-13 in patients with IgG4-RD (3.99 ± 6.15 pg/mL) were comparable to those in HC (3.18 ± 2.61 pg/mL; *P* = 0.524), and both were significantly lower than in patients with pSS (10.05 ± 13.99 pg/mL; Figure 5D). Serum levels of IL-10 in patients with IgG4-RD (10.67 ± 5.50 pg/mL) were significantly higher than those in HC (3.22 ± 1.40 pg/mL; *P* <0.001), and there were no significant differences between IgG4-RD and pSS patients (11.59 ± 7.90 pg/mL; Figure 5E). Serum levels of IFN-γ in patients with IgG4-RD (2.23 ± 1.56 pg/mL) were comparable to those in pSS patients (3.20 ± 3.15 pg/mL; *P* = 0.493), and both were significantly higher than in HC (1.02 ± 0.81 pg/mL; Figure 5F). As shown in Figure 5G, serum levels of BAFF in patients with IgG4-RD (2.60 ± 2.80 ng/mL) were significantly higher than those in HC (0.70 ± 0.42 ng/mL; *P* <0.001), while there were no significant differences in serum levels of BAFF between IgG4-RD and pSS patients (2.31 ± 2.65 ng/mL). Serum levels of IL-17A in patients with IgG4-RD (0.40 ± 0.57 pg/mL) were comparable to those in HC (0.24 ± 0.15 pg/mL; *P* = 0.482), and both were significantly lower than in patients with pSS (1.10 ± 1.37 pg/mL; Figure 5H). Serum levels of TGF-β in patients with IgG4-RD (16.94 ± 7.73 ng/mL) were comparable to those in HC (17.73 ± 4.81 ng/mL; *P* = 0.643), and both were significantly higher than in patients with pSS (14.16 ± 5.66 ng/mL; Figure 5I). In addition, there were no significant differences in the level of IL-5 in patients with IgG4-RD, pSS or HC (Figure 5C). Table 3 summarized cytokines expression in IgG4-RD, pSS patients and HC.

**Table 3:** Serum cytokine levels in IgG4-RD, pSS patients and HC

	**HC**	**IgG4-RD**	**pSS**	**IgG4-RD versus HC**	**pSS versus HC**	**IgG4-RD versus pSS**
	**(n = 24)**	**(n = 28)**	**(n = 28)**	***P-*** **value**	***P-*** **value**	***P-*** **value**
**IL-4, pg/mL**	1.69 ± 1.46	6.54 ± 5.67	6.52 ± 6.14	<0.001	<0.001	0.931
**IL-5, pg/mL**	1.34 ± 1.03	1.48 ± 1.51	2.23 ± 3.16	0.932	0.776	0.965
**IL-6, pg/mL**	0.68 ± 0.39	2.14 ± 1.66	1.35 ± 1.40	<0.001	0.002	0.009
**IL-10, pg/mL**	3.22 ± 1.40	10.67 ± 5.50	11.59 ± 7.90	<0.001	<0.001	0.697
**IL-13, pg/mL**	3.18 ± 2.61	3.99 ± 6.15	10.05 ± 13.99	0.524	0.016	0.013
**IL-17A, pg/mL**	0.24 ± 0.15	0.40 ± 0.57	1.10 ± 1.37	0.482	<0.001	0.001
**IFN-γ, pg/mL**	1.02 ± 0.81	2.23 ± 1.56	3.20 ± 3.15	0.002	0.002	0.493
**TGF-β, ng/mL**	17.73 ± 4.81	16.94 ± 7.73	14.16 ± 5.66	0.643	0.006	0.035
**BAFF, ng/mL**	0.70 ± 0.42	2.60 ± 2.80	2.31 ± 2.65	<0.001	<0.001	0.236

Values are shown as mean ± SD. IgG4-RD, IgG4-related disease; pSS, primary Sjögren’s syndrome; HC, healthy controls.

### Correlation of different B-cell subsets with laboratory findings in IgG4-RD and pSS patients

**Figure 6:**
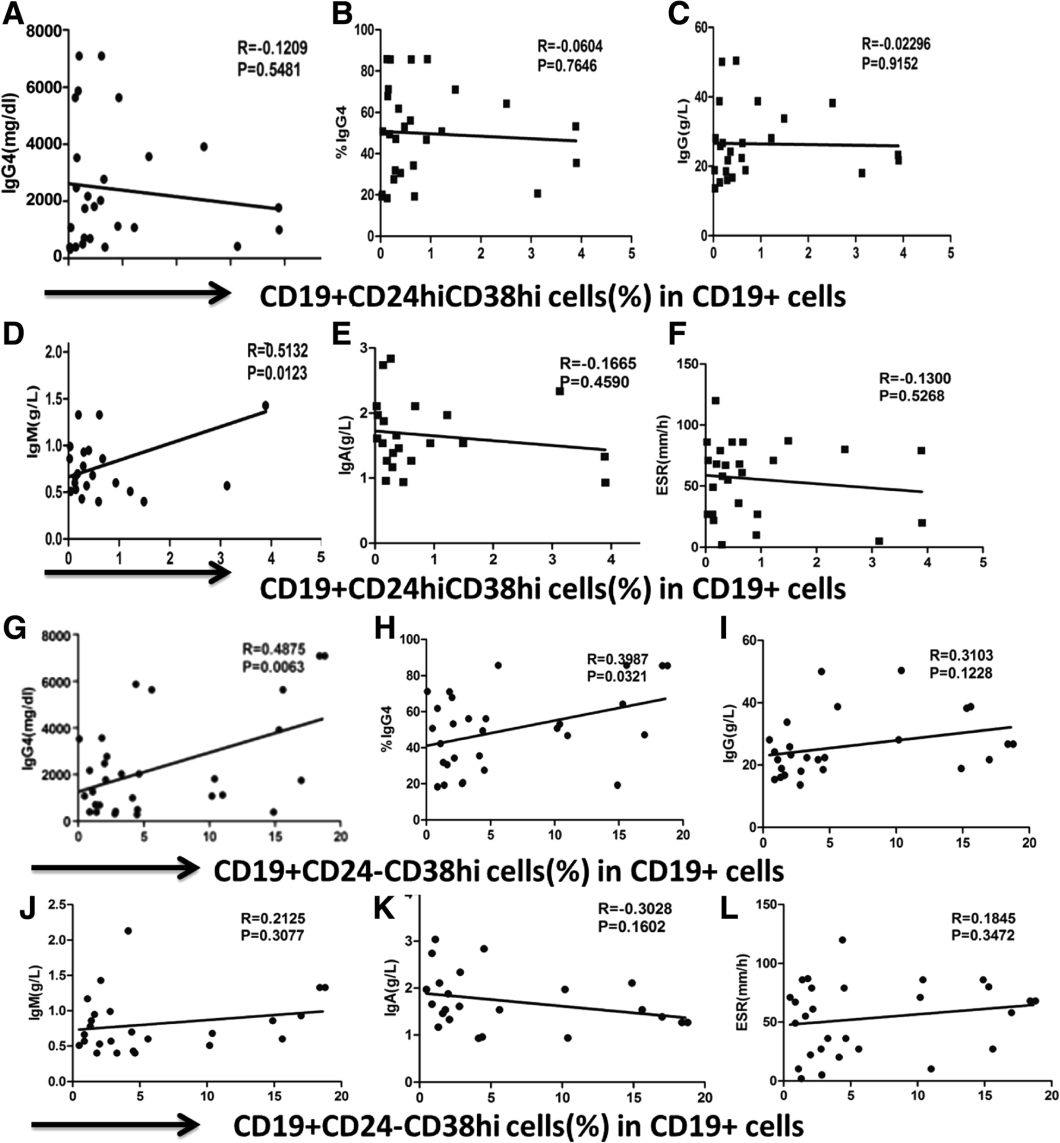
**Correlation of different B-cell subsets with laboratory findings in IgG4-related disease (RD) patients.** Correlations between the percentages of CD19 + CD24^hi^CD38^hi^ Breg cells and levels of IgG, IgM, IgA and IgG4, IgG4/IgG ratio, as well as erythrocyte sedimentation rate (ESR) in IgG4-RD patients (n = 28) were analyzed by Pearson’s rank test **(A-F)**. Correlations between the percentages of CD19 + CD24-CD38^hi^ B cells and levels of IgG, IgM, IgA and IgG4, IgG4/IgG ratio, as well as ESR in IgG4-RD patients were analyzed by Pearson’s rank test **(G-L)**.

The correlation of Breg cells with laboratory findings were evaluated in IgG4-RD and pSS patients. In IgG4-RD patients, there was no statistical correlation between the percentage of Breg cells and erythrocyte sedimentation rate (ESR), IgG, IgG4 levels, or IgG4/IgG ratio (Figure 6A-C). However, the frequencies of memory B-cell subsets were negatively correlated with serum IgG4, IgG levels and IgG4/IgG ratio in IgG4-RD patients (*r* = -0.5127, *P* = 0.004; *r* = -0.4759, *P* = 0.009; *r* = -0.4777, *P* = 0.014, respectively). Whereas there was no correlation between mature B cells, ESR, CRP, and serum immunoglobulin levels. As CD19 + CD24-CD38^hi^ B cells were significantly increased in IgG4-RD patients, we analyzed the correlation between percentages of CD19 + CD24-CD38^hi^ B cells and clinical findings. The frequencies of CD19 + CD24-CD38^hi^ B cells were positively correlated with IgG4 levels and the IgG4/IgG ratio in IgG4-RD patients (*r* = 0.4875, *P* = 0.0063; *r* = 0.3987, *P* = 0.0321; respectively; Figure 6G-H), indicating that CD19 + CD24-CD38^hi^ B cells might play an important role in IgG4-RD.

In contrast to IgG4-RD patients, we found that the frequency of Breg cells were correlated with ESR and serum IgG levels in pSS patients (*r* = 0.5292, *P* = 0.0032; *r* = 0.4376, *P* = 0.0109, respectively). However, there was no correlation between Breg frequencies and serum IgA and IgM levels. When observing other B-cell subsets, we found that the frequency of mature B cells was negatively correlated with ESR and serum IgG levels in pSS patients (*r* = -0.5042; *P* = 0.0038 and *r* = -0.3725; *P* = 0.0328, respectively). However, there was no correlation between memory B-cell frequencies, ESR, and serum immunoglobulin levels.

## Discussion

Regulatory B cells, as emerging immunoregulatory B-cell subsets, are considered an important regulator of the immune system [3,20,21]. Regulatory B cells are shown to play a vital role in various immune responses and diseases, including contact hypersensitivity and systemic autoimmune diseases. In this study, we tested the percentages of CD19+ B cells and its subsets, including regulatory, mature, and memory B cells in peripheral blood from IgG4-RD and pSS patients. According to Carsetti *et al*. and Blair *et al*., human Breg cells are a subset of B cells that express CD19 + CD24^hi^CD38^hi^[11,17]. Our data revealed that in contrast to HC, IgG4-RD patients had a significantly lower frequency of Breg cells, whereas pSS patients had a significantly higher frequency, although both diseases are characterized by hypergammaglobulinemia. We further tested the potential immune regulatory functions of Breg cells from pSS patients, which showed that unlike Breg cells from HC, CD19 + CD24^hi^CD38^hi^ Breg cells from pSS patients lack suppressive functions, as Breg cells were deficient in inhibiting proinflammatory cytokine production by Teff. The Breg function from IgG4-RD patients was not detected because of limited cell numbers. Similarly, Knippenberg and colleagues reported that the percentages of both memory B cells and Bregs from patients with stable and active multiple sclerosis were markedly decreased compared to HC [22].

CD40 signaling is critical for Breg cell generation and function in autoimmune disorders [23]. However, Blair *et al.* demonstrated that isolated CD19 + CD24^hi^CD38^hi^ B cells from systemic lupus erythematous (SLE) patients were refractory to further CD40 stimulation, produced less IL-10, and were deficient in suppressive capacity compared with their healthy counterparts [11]. In our study, CD40L-stimulated peripheral B cells from IgG4-RD patients did not induce an increase in Breg cells. One possible explanation is that for an unknown reason, Breg cells in IgG4-RD patients are not only deficient in number but also different in their response to CD40L stimulation.

Interestingly, we found a remarkable increase in the expression of a particular B-cell subset, which was characterized with CD19 + CD24-CD38^hi^ surface markers in peripheral B cells from IgG4-RD patients; this B-cell subset was expressed at fairly low levels in pSS and HC. In addition, CD19 + CD24-CD38^hi^ B cells positively correlated with serum IgG4 levels in IgG4-RD patients. According to a previous study, the CD19 + CD24-CD38^hi^ B cell subset comprised pre-germinal center (pre-GC) cells in tonsils, whereas there was no report of this B-cell subset in peripheral blood [19]. In IgG4-RD patients, the characteristic changes in involved tissues are massive infiltration of lymphocytes and IgG4+ plasma cells with GC formation in the majority of patients [24,25]. Therefore, we assumed that in IgG4-RD patients, the CD19 + CD24-CD38^hi^ B cells may differentiate into IgG4 producing plasma cells or recruit to the involved tissues. Further studies on this cell subset are needed.

It is well-established that BAFF, through BAFF-R, plays a key role in B-cell activation and survival [26,27]. It is reported that, over-expression of BAFF, participates in B cell abnormalities in pSS, such as aberrant B-cell distribution, B-cell hyperactivity, and autoantibody production [28,29]. As IgG4-RD patients are characterized with hypergammaglobulinemia and high serum IgG4 levels, we hypothesize that B cells from IgG4-RD patients might be in an endogenously activated state, with highly expressed BAFF-R, and readily differentiate into IgG/IgG4-secreting plasma cells. Although IgG4-RD shares many features with pSS, this study revealed that BAFF-R expression was significantly decreased in peripheral B cells in patients with IgG4-RD as compared with pSS and HC, whereas, the serum levels of BAFF in patients with IgG4-RD were significantly higher than those in HC. By stimulation with rhBAFF, the MFI of BAFF-R was decreased in B cells from healthy volunteers, consistent with the results of Sellam *et al.*, in which the decreased BAFF-R level was positively correlated with serum BAFF levels and associated with disease activity in pSS and SLE patients. Through post-transcriptional regulation, such chronic elevated overproduction of BAFF could downregulate BAFF-R expression on the B-cell surface, and BAFF-R level could be a novel activity biomarker in autoimmune diseases [30]. This may explain our findings that the same post-transcriptional regulation mechanism probably also occurred in IgG4-RD and reflects chronic BAFF overproduction.

Co-stimulatory factors play critical roles in T-cell and B-cell activation, wherein the CD40-CD40L and CD28-CD80/CD86 pathways are well known to cross-talk between T and B cells. CD40, a TNF superfamily transmembrane glycoprotein, plays an important role in B-cell differentiation and activation [31]. When activated with its ligand, CD40 provides a co-stimulatory signal which induces T cell-dependent B-cell proliferation and differentiation with subsequent immunoglobulin production. Ping *et al.* found that CD40 signals promote Fas-dependent death of pSS salivary epithelial cells by downregulating cellular FLICE-like inhibitory protein (c-FLIP) expression [32]. Our previous work has proved that CD40-induced nuclear factor (NF)-kB activation is different in human lupus B lymphocytes compared with normal B cells [33]. In addition, CD40 signaling is important for Breg cell production, as Breg cells can be induced by CD40 stimulation [34]. We evaluated CD40 expression in IgG4-RD, pSS and HC; the results not only revealed that in IgG4-RD patients CD19+ B lymphocytes expressed a lower frequency of CD40, but that decreased MFI of CD40 was also found in all B-cell subsets including regulatory, memory, and mature B cells from IgG4-RD patients. Furthermore, CD40 stimulation could not induce Breg cell expression. Therefore, our findings suggest that in IgG4-RD patients, CD40/CD40L signaling was aberrant in inducing Breg cell differentiation.

In contrast to CD40 expression, we found that both CD80 and CD86 expression on CD19+ B cells were significantly increased in patients with IgG4-RD, whereas their expression on CD19+ B cells was comparable between pSS and HC groups. Aberrant CD86 expression on B lymphocytes has been reported in many autoimmune diseases and T helper (Th)2 cells are polarized by CD86 [35]. Bijl *et al.* also showed the expression of CD86 on CD19+ B cells was increased and associated with B-cell activation [36]. In addition, Jeannin *et al.* proved that together with IL-4 or IL-13 and CD40L, CD86 was conducive to CD23-CD21 pairing, as the potential stimulating factor for human IgE and IgG4 synthesis [37]. It is encouraging that our data suggest that elevated CD86 and CD80 on B cells from IgG4-RD patients promote IgG4 and IgE synthesis. Moreover, previous studies have shown that the polarization of Th2 cells in IgG4-RD patients [6]. Consistent with these studies, our data showed Th2 polarization in IgG4-RD patients and Th2 cytokines, IL-4, but not IL-5 or IL-13, was significantly increased in IgG4-RD patients.

## Conclusions

Our data revealed that B-cell subsets were aberrant in both IgG4-RD and pSS patiens. However, B cells displayed differently in patients with IgG4-RD and pSS, including disturbed B-cell subpopulations and key signaling molecules, such as CD40, CD86, CD80 and BAFF-R. IgG4-RD patients had a lower frequency of regulatory and mature B cells but increased memory B cells compared with pSS patients and HC. Memory and CD19 + CD24-CD38^hi^ B cells were associated with IgG4 secretion. The function of the CD19 + CD24-CD38^hi^ B cell subsets in IgG4-RD remains to be elucidated. Whether aberrant expression of co-stimulatory molecules in patients with IgG4-RD correlates with class-switch needs further investigation.

## Abbreviations

APC: allophycocyanin; BAFF: B-cell activating factor; Breg: regulatory B cells; CBA: cytometric bead array; CRP: C-reactive protein; ELISA: enzyme-linked immunosorbent assay; ESR: erythrocyte sedimentation rate; FACS: fluorescence-activated cell sorter; FCS: fetal calf serum; HC: healthy controls; IFN: interferon; Ig: immunoblobulin; IgG4-RD: IgG4-related disease; IL: interleukin; mAbs: monoclonal antibodies; MFI: median fluorescence intensity; PBMC: peripheral blood mononuclear cell; pSS: primary Sjögren’s syndrome; RPMI: Roswell Park Memorial Institute; SEM: standard error of the mean; TACI: transmembrane activator and calcium modulator; Teff: effector T cells; TGF: transforming growth factor; Th: T helper; TNF: tumor necrosis factor.

## Competing interests

The authors declare that they have no competing interests.

## Authors’ contributions

WL, LJ, HC: data collection and analysis, experiments performed, manuscript writing, critical revision and final approval of the manuscript. QWu, YF, QWa, WZe: data collection, manuscript writing, critical revision and final approval of the manuscript. WZa, XZ: conception and design, data collection and analysis, critical revision and final approval of the manuscript. YZ, FZ: conception and design, data collection, financial support, critical revision and final approval of the manuscript. YL, PL: data collection, financial support, critical revision and final approval of the manuscript. All authors read and approved the final manuscript.
